# Entropy-Based Risk Control of Geological Disasters in Mountain Tunnels under Uncertain Environments

**DOI:** 10.3390/e20070503

**Published:** 2018-07-01

**Authors:** Yuanpu Xia, Ziming Xiong, Zhu Wen, Hao Lu, Xin Dong

**Affiliations:** 1 State Key Laboratory of Disaster Prevention & Mitigation of Explosion & Impact, the Army Engineering University of PLA, Nanjing 210007, China; 2 School of Mechanical Engineering, Nanjing University of Science and Technology, Nanjing 210094, China

**Keywords:** uncertainty, risk control, decision-making, tunnel engineering, entropy

## Abstract

Uncertainty is one of the main sources of risk of geological hazards in tunnel engineering. Uncertainty information not only affects the accuracy of evaluation results, but also affects the reliability of decision-making schemes. Therefore, it is necessary to evaluate and control the impact of uncertainty on risk. In this study, the problems in the existing entropy-hazard model such as inefficient decision-making and failure of decision-making are analysed, and an improved uncertainty evaluation and control process are proposed. Then the tolerance cost, the key factor in the decision-making model, is also discussed. It is considered that the amount of change in risk value ($R_{1}$) can better reflect the psychological behaviour of decision-makers. Thirdly, common multi-attribute decision-making models, such as the expected utility-entropy model, are analysed, and then the viewpoint of different types of decision-making issues that require different decision methods is proposed. The well-known Allais paradox is explained by the proposed methods. Finally, the engineering application results show that the uncertainty control idea proposed here is accurate and effective. This research indicates a direction for further research into uncertainty, and risk control, issues affecting underground engineering works.

## 1. Introduction

Tunnelling is an important part of infrastructure projects that can improve connections, improve the quality of life and promote economic development. Their benefits cannot be over-stated, and the world is witnessing an ever-increasing need for tunnels [1,2]. Since geotechnical media are materials that can change instantly and significantly from one point to the next [3], and tunnels are large-scale complex systems, various uncertainties are involved throughout the whole construction period. Therefore, risk will also accompany the entire construction cycle, which will affect all parties involved, and even those not directly involved, in such projects [4,5]. For tunnel engineering in complex geological areas, such as in the southwest mountainous region of China with high geo-stress, high water pressure, and karst features, such projects face serious geological disaster risk (e.g., from water inrush [6], collapse [7], rock burst [8], tunnel face instability [9,10], etc.) when tunnelling. When these disasters occur, it not only causes delays in construction, economic losses, but also causes casualties [11]. Due to the widespread existence of various types of risks mentioned above, overruns and delays are widespread in tunnel engineering [12,13]. How to deal with relevant uncertainties and control risk within an acceptable level with reasonable time, cost and limited resources, which is not only an important concern, but also a difficult issue in tunnel engineering and academic circles.

**Figure 1:**
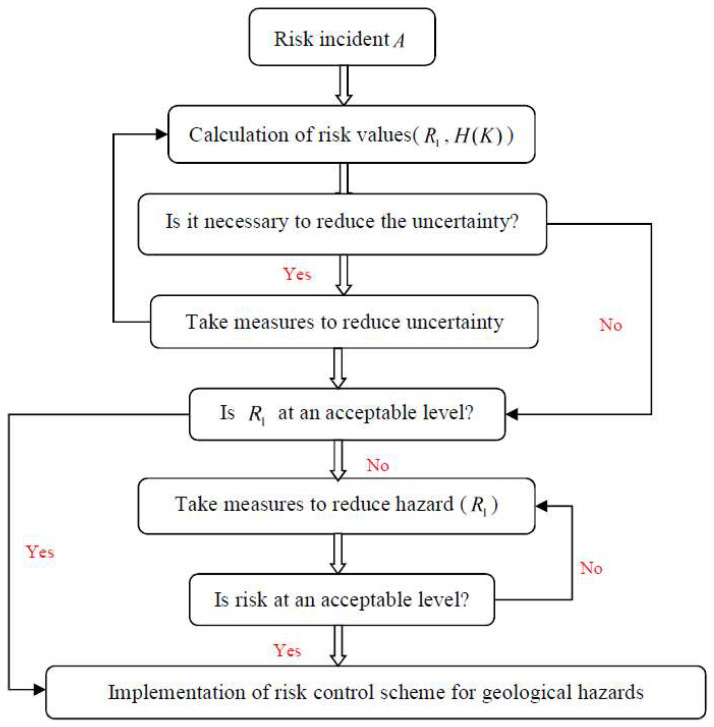
Risk control for geological hazards in tunnel engineering.

The relationship between uncertainty and risk and its impact on risk analysis has been discussed by many scholars, such as Aven [14] who proposed that using (C, U) instead of (C, P), where “C” represents the possible consequences, p represents the probability of risk event and“U” indicates uncertainty lead to a risk description associated with the definition of (C, U) as ($C^{\prime }$, Q, K), where $C^{\prime }$ denotes specified consequences; Q is a measure of uncertainty such as probability [15,16], imprecise probability [17], or a fuzzy number [18,19]; and K can be considered to represent background knowledge on which the specifications and assignments of $C^{\prime }$ and Q are based [20,21]. For the new definition of risk, it can be seen that the main contents of risk research is: ($R_{1}=C\times Q$, $R_{2}=H(K)$), where $R_{1}=C\times Q$ represents risk assessment value, i.e., hazard, $R_{2}=H(K)$ representing entropy was used to quantify the degree of uncertainty of background knowledge. Therefore, risk control should also be considered mainly from the following two perspectives: (1) how to reduce the degree of uncertainty H(K), and (2) how to reduce the hazard (); however, uncertainty and hazard are not independent. The changes in H(K) are likely to cause changes in *R*_1_, and the dynamic relationship between them makes risk-control more complicated. By considering these two factors, we constructed a reasonable risk control process (Figure 1).

From Figure 1, we can see that the risk control process mainly involves risk assessment and risk decision-making. The purpose of risk assessment is to provide reliable information for decision-making with regard to choosing reasonable risk control measures. Current research on risk control in tunnel engineering mainly focusses on risk assessment and selection of hazard control measures. For example, to control the risk of water inrush, various risk assessment models are proposed [22,23,24,25,26]; however, the impact of uncertainty on risk control has not yet been paid sufficient attention. From Figure 1, uncertainties will affect the reliability of assessment results and further affect the rationality of decision-making results. In actual projects, disasters caused by the irrationality of risk control programmes are also an important part of safety engineering. For example, due to the unreasonable processing of uncertainty, the risk control scheme may not match the risk, there will be risk control failures or risk control measures may be too conservative. The former may lead to disaster, while the latter will lead to a waste of resources, time, etc.

To improve the effectiveness of risk control, it is necessary to describe objectively the uncertainty and reduce the impact of uncertainty on assessment and decision-making. The former has been widely discussed, but the latter has not attracted enough attention. Dong et al. [27] and Xia et al. [28] based on the characteristics of tunnel engineering, the latest risk theory and the advantages of entropy to quantify uncertainty information [29], the entropy-hazard model was constructed to discuss the risk control problem under uncertain information. The target is to assess the impact of uncertainty and determine whether measures needed to reduce uncertainty, which represents significant progress for risk control in tunnel engineering; however, there are still some deficiencies in the related research results, mainly including: (1) as there are many factors that affect the uncertainty, the available measures to reduce the uncertainty will be much, and there will be blind to decision-making by these models; (2) control measures only considering cost may be unreasonable, as other factors such as time and the environment may also affect decisions; and (3) the relationship between tolerance costs $T_{H}^{\prime }$, uncertainty H(K), and hazard ($R_{1}$) needs further study. These deficiencies lead to poor operability of the model in practice.

This paper focuses on the above problems and further improves the model so as to improve the reliability and decision-making efficiency. It is organised as follows. In Section 2, we mainly discuss and perfect the limitations in the entropy-hazard model, and propose a reasonable uncertainty assessment and control method. Section 3 introduces an improved processing method to overcome the limitations of existing multiple attribute decision-making methods under uncertain environments. In Section 4, the issues of uncertainty in assessment and control of risk in Zhiziyuan tunnel is analysed and discussed based on the improved model, and the rationality and engineering adaptability of the model are verified. Finally, Section 5 provides our conclusions.

## 2. Risk Control Considering Uncertainty

Elsewhere [28], a preliminary framework has been proposed to guide the reduction of uncertainty; however, in practical applications, we found some deficiencies as mentioned in the introduction.

(1) The Computational Efficiency of the Model

The reduction of uncertainty is indeed important for risk control, but taking into account various factors such as cost and time, it is unrealistic to completely eliminate the uncertainty in the application. It is our goal to control the impact of uncertainty on assessment and decision-making to within an acceptable range. For tunnel engineering geological disaster risk, due to the variety of sources of uncertainty, the factors affecting the uncertainty may be as many as a dozen, but we only need to consider taking measures to reduce uncertainty in one, or several, thereof. In addition, we can take different measures even for the same factor. Therefore, it will lead to a very large number of alternatives, which not only increases the computational burden and difficulty of selection, but also easily leads to situations where multiple schemes meet the requirements. We now provide further explanation by enumerating a simple example similar to that in the literature [27].

According to the statistical analysis of historical data, the fitting formula between the occurrence probability of incident A and the influencing factors is:

(1) $$P(A)=0.1x_{1}+0.02x_{2}^{2}+0.09x_{2}+0.05x_{3}+0.02x_{4}+0.03x_{5}^{2}+x_{1}x_{4}$$

There are five influencing factors ($x_{1},x_{2},x_{3},x_{4},x_{5}\in (0,1)$) in the above formula, the scope of risk control measures will be very wide. It will be a challenging issue to select a reasonable control measure quickly. The existing model is inefficient and has some blindness.

To compensate for the above deficiencies, we believe that sensitivity analysis is necessary for all uncertain variables (influencing factors) to determine the impact of each variable on hazard (*R*_1_). Only those variables with a larger influence will be considered. This preliminary judgment can improve the efficiency of decision-making and reduce blindness.

(2) The Relationship between Hazard and Tolerance Cost $T_{H}^{\prime }$

Different combinations of uncertainties and hazards will lead to different choices for risk control. Table 1 lists four typical combinations quoted from the literature [28].

For the first situation, no risk control measures will be taken, and in the second case we will choose measures to reduce the hazard. What they have in common is that they do not need to take measures to reduce uncertainty. We can think that their tolerable costs should be closer ($T_{H}^{\prime }(1)\approx T_{H}^{\prime }(2)$, not too far apart; however, according to the calculation model (Formula (2), where $e_{\max}$ indicates ”decision effect”, and α is an undetermined coefficient with α>0 [27]), the tolerable costs obtained may vary greatly and the above phenomenon cannot be fully reflected. Similarly, for the third and fourth situations, we are all willing to provide more resources and cost to deal with the third type of situation, because it is not only of large uncertainty, but also hazardous.

(2) $$T_{H}^{\prime }=\alpha H_{\max}e_{\max}\cdot \max(R_{1},R_{1}^{\prime })$$

In an ideal state, if the uncertainty is zero, the hazard will be a fixed value, but for tunnel engineering, because of uncertainty, such as uncertainty of geological information and uncertainty of disaster mechanism, the value of hazard is uncertain. Now we assume that there are events A and B, the degrees of uncertainty are the same, that is, H(A)=H(B), and suppose that the maximum hazard value is 100, then according to the existing uncertain information, the hazards are: $R_{1}(A)=[20,80]$, $R_{1}(B)=[70,80]$, respectively. For event A, we will choose to reduce uncertainty to determine the range of hazard values: for event B, because the range of hazard values is relatively small, it will be more effective for risk control to take measures to reduce the hazard. Therefore, for case where the hazard is uncertain, the tolerance cost is not related to the hazard value, but to its range, that is $\Delta R_{1}$. Therefore, the following formula may be more reasonable:

(3) $$T_{H}^{\prime }=\alpha H_{\max}e_{\max}\cdot \Delta R_{1}$$

(3) Determination of Risk Control Scheme

In the existing method, the control scheme is determined when judging whether or not it is necessary to reduce the uncertainty. That is, the formula $T_{H}\leq u(T_{H}^{\prime })$ has played two roles, namely, necessity judgment and scheme selection. However, in practical applications, two or more schemes may meet the requirements. Assuming a utility tolerable cost is $u(T_{H}^{\prime })=1800$, the implementation costs of the scheme A and B are: $T_{H}(A)=1720,\;T_{H}(B)=1690$, respectively. Then how do we choose? If we do not consider other factors, we should choose B, which has the lower cost. We now assume that implementation of A will take less time, and its impact on environment, such as water pollution, will be relatively small. Under this situation, will we still insist on choosing B? Therefore, it may be not appropriate to consider only the decision effect, other main factors should also be considered. It will be a multi-attribute utility decision problem under uncertainty and how to choose will be discussed below.

Through the above analysis, it can be seen that the existing entropy-hazard model needs to be improved when used in tunnel engineering. With regard to how to analyse, and reduce, the impact of uncertainty on risk, we believe that the following steps are more reasonable:

**Step 1:** Use entropy to calculate the sensitivity of variables, and narrow the scope of alternatives according to sensitivity, so as to improve decision-making efficiency.

The main purpose of sensitivity analysis (SA) is to estimate effects of each model input on the model response and to identify the primary contributors to output uncertainty [30], which could increase the understanding of the relationships between input variables and the output [31]. Then the variables that cause significant variability in the output results should be the focus [32].

Variance-based SA [33] is the most commonly used method. In addition, there are other SA methods, such as Kullback–Liebler divergence methods, screening methods, entropy-based SA, etc. [34,35]. In recent years, as it could reveal complementary or additional information compared to the most often variance-based method, entropy-based SA methods have received more attention [36]. Therefore, we try to quantify the impact of input variables on the risk of geological hazards by the entropy-based SA.

Suppose there is a limit state function: Y=f(X), where $X=\{x_{1},x_{2},\cdots ,x_{n}\}$ represents random input variables, i.e., model parameters, f(X) is the response function, Y are the output results. So the sensitivity index formula can be as follows:

(4) $$\eta_{x_{i}}=\frac{\left|H[u_{y}]-H[u_{y|x_{i}}]\right|}{H[u_{y}]}$$

where $H[u_{y|x_{i}}]$ indicates the degree of uncertainty of the model output without considering $x_{i}$.

**Step 2:** The necessity of reducing uncertainty.

The object of this step is to determine the tolerable cost. If Formula (3) is taken, the tolerance cost is different for each scheme, but for the same risk event, the tolerance cost is relatively fixed for decision-maker, which is also consistent with decision-making habits in tunnel engineering. Therefore, Formula (3) is further modified to get Formula (5), and the decision effect (e) is considered together with other factors such as time, environment, etc. as influencing factors.

(5) $$T_{H}^{\prime }=\alpha H_{\max}\Delta R_{1}$$

**Step 3:** Decision-making with different attributes.

To obtain a reasonable decision-making result, not only attribute information, but also information uncertainty, should be considered. In addition, the attitude of decision-makers to risks can also not be ignored.

For tunnel engineering, the analysis process for such uncertainty is as follows:

## 3. Scheme Selection under Multi-Source Attributes

**Figure 2:**
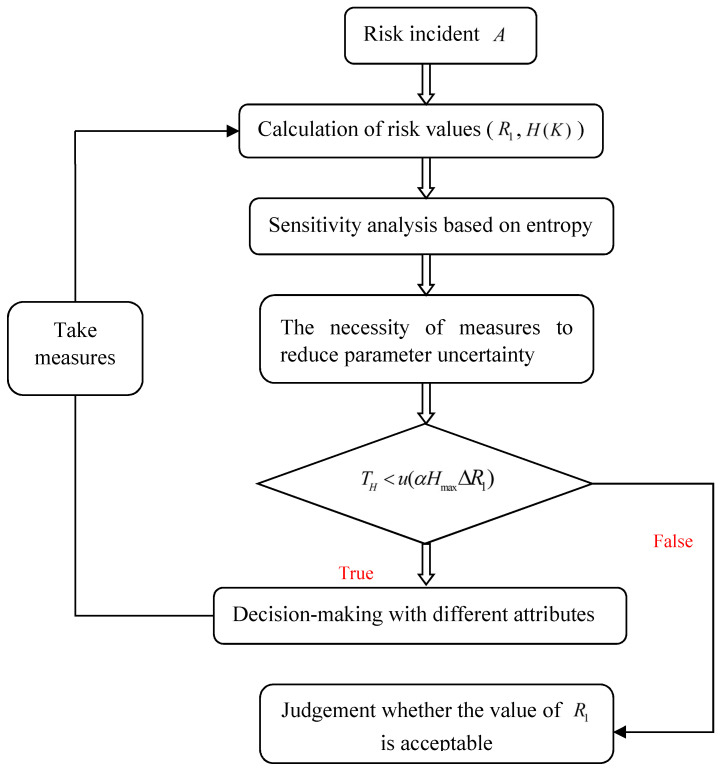
Analysis process of uncertainty on risk control in tunnelling works.

As shown in Figure 2, for the schemes that meet the tolerable cost, we need to optimise the schemes in terms of properties such as time, environmental impact, application effect, and then choose the most reasonable scheme. The impact of attributes on schemes can be viewed as a consequence of risk. Since the attribute information is uncertain, according to the definition of risk, risk and uncertainty must be considered simultaneously when decision-making. Therefore, it is a typical multi-attribute decision-making problem under risk and uncertainty, which has aroused wide interest of scholars from different industries. For example, in order to promote the research of managing information uncertainty and complexity in decision-making, Antucheviciene et al. [37] initiated related special issue research, and got the active response from scholars with different industries. Since the decision-making issue is more and more complex in civil engineering, and the characteristics of uncertainty are very obvious, in recent years, the study of multi-criteria decision making has been becoming more and more popular in civil engineering. Such as Antucheviciene et al. [38] have systematically summarised and analysed the research status of MADM in civil engineering from the following aspects, including the influence of uncertainty, the decision-making problem under risk, the necessity of sensitivity analysis etc, and put forward several problems that need further study in the future. To solve the problems of multi-attribute decision making under uncertainty, Yang et al. [39] first proposed their expected utility-entropy (EU-E) model. Although the EU-E model has been applied in different fields, such as a decision-making model for large consumers on a smart grid [40], rainfall threshold analysis [41], stock selection [42], etc., few people could question the rationality of the EU-E model itself. Fischera [43] commented on Yang’s model and pointed out its deficiencies, such as that the impact of consequences was not considered for uncertainty analysis; however, it does not propose a reasonable alternative framework or model, but simply thinks that the EU model is enough to solve decision-making problems under uncertainty. Therefore, based on the existing research, we analyse the EU-E model, and corresponding improvement measures are proposed. Yang’s EU-E model is defined as follows:

(6) $$R(a)=\lambda H_{a}(\theta )-(1-\lambda )\frac{E[u(X(a,\theta ))]}{\max\{E[u(X(a,\theta ))],a\in A\}}$$

where, X(a,θ) is the outcome resulting from the combination of option a and state θ (such as a can be numbers, and θ indicates corresponding probability), u(X) is the decision-maker’s utility function; the factor λ with 0≤λ≤1 expresses the weighting, or trade-off, that the decision-maker attaches to the expected utility and entropy. Some examples have been proposed in [39,43] to prove or refute the reliability or deficiencies of the EU-E model, but they fail to consider all possible situations. To analyse the reliability of the EU-E model under different circumstances, based on the difference in entropy values, the decision-making situations are divided into the following three categories (for the sake of this analysis, we only consider the situation with two schemes a and b):

(1) The uncertainty of the consequences is equal, H(a)=H(b), then we can further divide it into three situations.

① H(a)=H(b), E(a)=E(b), E(a), E(b) are expected utility, and the related example is as follows:

If scheme a is (2, 1/3; 4, 1/3; 6, 1/3), where 2, 4, 6 are numbers; 1/3 represents the corresponding probability, and the follow-up cases are similar to a, then scheme b can be $b_{1}$: (0, 1/3; 1, 1/3; 11, 1/3), $b_{2}$: (0, 1/3; 2, 1/3; 10, 1/3), $b_{3}$: (0, 1/3; 3, 1/3; 9, 1/3), $b_{4}$: (0, 1/3; 4, 1/3; 8, 1/3), $b_{5}$: (0, 1/3; 5, 1/3; 7, 1/3), $b_{6}$: (1, 1/3; 2, 1/3; 9, 1/3), $b_{7}$: (1, 1/3; 3, 1/3; 8, 1/3), $b_{8}$: (1, 1/3; 4, 1/3; 7, 1/3), or $b_{9}$: (3, 1/3; 4, 1/3; 5, 1/3).

If Formula (6) is used to determine which scheme should be selected, we have R(a)=R(b), the EU-E model is invalid at this time; because the value of λ reflects the decision-maker’s attitude to risk, where 0≤λ<0.5 this indicates decision-makers are risk averse, λ=0.5 means risk neutrality, and 0.5<λ≤1 indicates that the decision-maker is willing to take a greater risk. So different values of λ should be considered.

For the risk-averse, the worst consequence of scheme a is 2. Compared to schemes $b_{1}$ to $b_{8}$, a is more reasonable. For schemes a and $b_{9}$, $b_{9}$ is obviously more reasonable. Since the order of variances is Var(b_9_) < Var(a) < Var(b_1_~b_8_), a reasonable decision can be made based on variance.

For decision-makers with a risk-neutral view (λ=0.5), since there is no obvious risk tendency, it is difficult to make a decision between schemes a and b. No matter which is chosen, decision-makers can give their reasons, but intuitively, we believe it is more likely to have the same decision-result as a risk-averse manager.

Since risk seekers are willing to take higher risks for a better outcome, compared to scheme a, schemes $b_{1}$ to $b_{8}$ seem more reasonable. For schemes a and $b_{9}$, decision-makers are more likely to choose a.

Even when faced with the same risk decision event, decision-makers will make different decisions due to differences in their attitudes.

② H(a)=H(b) and E(a)<E(b), the related example is as follows:

If scheme a is (2, 1/3; 4, 1/3; 6, 1/3), as there are many schemes of b that could satisfy the conditions H(a)=H(b) and E(a)<E(b), we select several representative examples, such as $b_{1}$: (0, 1/3; 1, 1/3; 17, 1/3), $b_{2}$: (6, 1/3; 9, 1/3; 12, 1/3), $b_{3}$: (10, 1/3; 11, 1/3; 12, 1/3).

According to Function (6), R(a)>R(b), however, decision-making results must also consider the influence of attitude to risk.

For the risk-averse, compared to $b_{1}$, scheme a is a relatively ideal result, but if it is compared to $b_{2}$ or $b_{3}$, it would be more reasonable to choose $b_{2}$ or $b_{3}$. Therefore, for such problems, neither the use of an EU-E model nor the variance can reach reasonable judgments.

For decision-makers with risk seeking behaviour, schemes $b(b_{1},b_{2},b_{3})$ would be the ideal choice, if compared to scheme a, and the EU-E model is effective.

For decision-makers who are risk-neutral (λ=0.5), since the expected value E(a)<E(b), scheme b is more reasonable, and the EU-E model is effective.

The EU-E model is effective in some cases, but there is no discussion of this in the literature [37].

③ For the case where H(a)=H(b) and E(a)>E(b), the analysis process and results are the same as the second type (H(a)=H(b), E(a)<E(b)) and are not described here.

(2) The uncertainty of the consequence for scheme a is larger than b, e.g., H(a)>H(b). Then we can further divide it into three situations.

① H(a)>H(b), E(a)=E(b), then R(a)>R(b), (R(a)=R(b), if λ=0) can be obtained based on Function (6), the related example are as follows.

Scheme a: (2, 1/4; 4, 1/4; 6, 1/4; 8, 1/4); Scheme b: (10−x, 1/2; x, 1/2), (5<x≤10) and H(a)=1.39, H(b)=0.69, E(a)=E(b)=5, according to Function (6), assuming λ≠0, we have R(a)=1.39λ−(1−λ)>R(b)=0.69λ−(1−λ). Now we let x=10,8,6, respectively. So scheme $b_{1}$: (0, 1/2; 10, 1/2); scheme $b_{2}$: (2,1/2; 8, 1/2); and scheme $b_{3}$: (4, 1/2; 6, 1/2). Since different values of λ represent different attitudes to risk of the decision-maker, it is more reasonable to analyse this case considering these attitudes.

For the risk-averse, schemes a and $b_{1}$ are offered, since scheme $b_{1}$ has one-half probability with consequence of 0, mean that they are intuitively more inclined to choose scheme a. For schemes a and $b_{2}$, the latter has a higher probability of 2 than scheme a, and for risk conservatives, the risk of choosing a is relatively small. For schemes a and $b_{3}$, the latter is more likely to take 4, and does not take the risk of value 2, so it is more reasonable to choose scheme $b_{3}$. Here, the EU-E model is invalid, but the variances of $a,b_{1},b_{2}$ and $b_{3}$ are Var(a)=5, $Var(b_{1})=25$, $Var(b_{2})=9$, and $Var(b_{3})=1$, respectively, so in these situations, a reasonable choice can be made based on the variance.

For decision-makers with a risk-neutral view (λ=0.5), since they do not have a clear tendency toward risk, it is difficult to make judgments for the above situations. Regardless of whether choosing $a,b_{1},b_{2}$ or $b_{3}$, there are reasons for their rationality, but intuitively, the results of the choice are more likely to be consistent with a risk-averse manager.

For decision-makers with risk seeking behaviour, decision-makers are willing to take higher risk. So for schemes a and $b_{1}$, a and $b_{2}$, it will be more reasonable to choose $b_{1}$ and $b_{2}$. For schemes a and $b_{3}$, we are more inclined to choose scheme a.

② H(a)>H(b), E(a)>E(b), the values of R(a) and R(b) cannot be compared directly with Equation (6).

**The first situation**: R(a)=R(b), the decision result cannot be obtained based on EU-E model. If scheme a is (2, 1/4; 4, 1/4; 6, 1/4; 8, 1/4), then scheme b can be (0, 1/2; x,1/2), ($x=10(1-\frac{0.7\lambda }{1-\lambda })$, 0<λ<0.5882 for x>0, E(a)>E(b) and R(a)=R(b)). 0<λ<0.5882 indicates that the decision-maker is not absolutely risk-seeking, and is more likely to be conservative or neutral. Assuming that the decision-maker is risk neutral, λ=1/2, then scheme b is (0, 1/2; 8.6, 1/2). According to the variance: Var(a)=5<Var(b)=18.5, we should choose a, which is consistent with the attitude to risk of the decision-maker. For the risk-averse, scheme a is more reasonable.

**The second situation**: R(a)>R(b). If scheme a is (2, 1/4; 4, 1/4; 6, 1/4; 8, 1/4), then scheme b can be (0, 1/2; x, 1/2), (0<x<10, $x>10(1-\frac{0.7\lambda }{1-\lambda })$, 0<λ<1 for E(a)>E(b) and R(a)>R(b)). When 0<λ<0.5882, $10(1-\frac{0.7\lambda }{1-\lambda })>0$, then we get $10(1-\frac{0.7\lambda }{1-\lambda })<x<10$; when 0.5882<λ<1, $10(1-\frac{0.7\lambda }{1-\lambda })<0$, then we have 0<x<10.

For the risk-averse, such as those for whom λ=0.1, then 9.22<x<10. If scheme $a_{1}$ is (2, 1/4; 4, 1/4; 6, 1/4; 8, 1/4), b is (0, 1/2; 9.5, 1/2), then scheme a should be chosen. Now we let scheme $a_{2}$ be (0, 1/4; 1, 1/4; 2, 1/4; 17, 1/4), and b remains unchanged, we will be more inclined to choose scheme b. The order of variance is $Var(a_{1})=5<Var(b)=22.56$, $Var(b)=22.56<Var(a_{2})=48.5$. Therefore, for this situation, it is reasonable to make decisions based on variance.

For a risk-neutral decision-maker (λ=0.5, 3<x<10), it is difficult to make a reasonable decision. Now let scheme b be $b_{1}$: (0, 1/2; 4, 1/2); $b_{2}$: (0, 1/2; 5, 1/2); $b_{3}$: (0, 1/2; 6, 1/2); $b_{4}$: (0, 1/2; 7, 1/2); $b_{5}$: (0, 1/2; 8, 1/2); $b_{6}$: (0, 1/2; 9, 1/2). Comparing $b_{1},b_{2}$ and $b_{3}$ to scheme a (2, 1/4; 4, 1/4; 6, 1/4; 8, 1/4), then scheme a is more reasonable. For schemes $b_{4}$ and a, it does not violate basic decision-making behaviour if choosing a or $b_{4}$. We believe that alternatives $b_{5}$ and $b_{6}$ are more likely to be selected than a. Therefore, neither the expected EU-E model nor the variance are applicable. It is rather complicated for this situation and it needs to be analysed according to the specific situation.

For a decision-maker seeking risk, such as at λ=0.9, 0<x<10, if 0<x≤6, the probability that the consequence of scheme a exceeds 6 is 50%, then the decision-makers are more inclined to scheme a. If 6<x<10, decision-makers will choose scheme b to pursue higher reward.

**The third situation**: R(a)<R(b). We still let scheme a have (2, 1/4; 4, 1/4; 6, 1/4; 8, 1/4), then scheme b can be (0, 1/2; x, 1/2), ($0<x<10(1-\frac{0.7\lambda }{1-\lambda })$, 0<λ<0.5882 for E(a)>E(b) and R(a)<R(b)). In such a situation, it is more reasonable for decision-makers who are risk-averse or risk-neutral.

For the risk-averse, scheme a is an ideal goal. For a risk-neutral decision-maker (λ=0.5, 0<x<3), scheme a should also be chosen and, in this case, the EU-E model is effective.

③ H(a)>H(b), E(a)<E(b). Then according to Formula (6), we have R(a)>R(b). Assuming scheme a is (2, 1/4; 4, 1/4; 6, 1/4; 8, 1/4), there will be many types of scheme b, such as (0, 1/2; x, 1/2), (x>10, 0<λ<1 for E(a)<E(b) and R(a)>R(b)). The risk attitude of decision-makers is uncertain.

Now we let scheme b be $b_{1}$: (0, 1/2; 20, 1/2), $b_{2}$: (1, 1/2; 19, 1/2), $b_{3}$: (2, 1/2; 18, 1/2), $b_{4}$: (4, 1/2; 16, 1/2), $b_{5}$: (6, 1/2; 14, 1/2), $b_{6}$: (8, 1/2; 12, 1/2). If we compare $b_{1},b_{2}$ to a, since $b_{1}$ has the possibility of consequence of zero, and $b_{2}$ offers the possibility that the consequence is smaller than that of scheme a, for the risk-averse, it is more stable to choose scheme a. However, when compared to $b_{3}$ to $b_{6}$, since the minimum possible outcomes of the latter are equal to, or greater, than the possible value of scheme a, so it is more consistent with the decision-making behaviour of a risk-averse manager to choose $b_{3}$ to $b_{6}$. If we have a: (0, 1/4; 1, 1/4; 2, 1/4; 17, 1/4), b: (0, 1/2; 20, 1/2), since both schemes have the possibility of zero consequence, that is, the worst result is the same, then in this case, for the risk-averse, they will choose a scheme that has a larger expected utility value, that is, scheme b.

For a risk-neutral decision-maker (λ=0.5), as decision-makers have no obvious risk tendencies, it is difficult to judge whether to choose scheme a or b, but we can confirm that with the increasing value of *x*, the probability of selecting scheme b will increase. It can be considered to be similar to a risk-averse decision-making behaviour.

For a risk-seeking decision-maker, to pursue higher consequences, scheme b is an ideal goal. In this case, the EU-E model is effective.

(3) The uncertainty of the consequence for a is smaller than b, e.g., H(a)<H(b). Then we can further divide it into three situations.

① H(a)<H(b), E(a)=E(b), then we have R(a)<R(b), (0<λ<1). The principle of analysis is the same as the situation whereby H(a)>H(b), E(a)=E(b). For example, if we have scheme a: (10 − x, 1/2; x, 1/2), scheme b: (2, 1/4; 4, 1/4; 6, 1/4; 8, 1/4), then the analysis result is the same as that with H(a)>H(b), E(a)=E(b).

Similarly, for case H(a)<H(b), E(a)<E(b), the result is the same as that with H(a)>H(b), E(a)>E(b). For case H(a)<H(b), E(a)>E(b), the result is the same as that with H(a)>H(b), E(a)<E(b).

Through the above analysis, we can see that for multi-attribute decision-making issues under an uncertain environment, it could not solve all situations by simply relying on the expected utility-entropy model. It not only needs to consider the expected utility, uncertainty measure, variance, etc., but more importantly, the influence of the risk attitude of the decision-maker cannot be ignored, different risk attitudes will lead to different results. Assume that there are two schemes a and b, the minimum value and its probability, the maximum value and its probability in each scheme are: ($a_{\min},l$), ($b_{\min},m$), ($a_{\max},p$), ($b_{\max},q$), respectively. Based on the above analysis, we recommend discriminant methods for the different types of scheme combinations in Table 2.

The decision problem is fundamentally a subjective judgment of the decision-maker. Therefore, subjective factors affecting a decision-maker are important. Due to external environmental factors and their own knowledge level, the decision result is often a bounded rational entity. Decision-making issues must take into account the risk attitudes (Table 2), different risk attitudes exert a decisive influence on decision-making results. The EU-E model or variance-average model can only solve some parts of the problems. By classifying decision-making problems, it is more reasonable to propose different methods according to different situations.

The well-known Allais paradox (Table 3) has been discussed elsewhere [39] to prove the validity of EU-E model. We will analyse the paradox based on the proposed method.

**Problem 1**: Select $a_{1}$ or $b_{1}$?

The entropy and expected values are: $H(a_{1})=10$, $H(b_{1})=0.38$, $E(a_{1})=10$, $E(b_{1})=1.39$, $R(a_{1})=-0.72(1-\lambda )$ and $R(b_{1})=1.38\lambda -1$. When 0.42<λ≤1, then $R(a_{1})<R(b_{1})$, so the $a_{1}$ should be chosen, and Yang believes the EU-E model is reasonable; however, why is it not considered with 0≤λ≤0.42? Now we analyse the issue by the proposed method: as $H(a_{1})<H(b_{1})$, $E(a_{1})<E(b_{1})$, according to Table 3, we know that there will be three cases: $R(a_{1})=R(b_{1})$, $R(a_{1})>R(b_{1})$ or $R(a_{1})<R(b_{1})$.

$R(a_{1})=R(b_{1})\Rightarrow \lambda =0.42$, which means that decision-maker is biased towards risk conservatism. Using Table 3, the result can be obtained based on variance. Since $Var(a_{1})<Var(b_{1})$, we should choose $a_{1}$.

$R(a_{1})>R(b_{1})\Rightarrow \lambda <0.42$, which indicates risk-aversion; the variance is the recommended method in Table 3 and $a_{1}$ is our option. $R(a_{1})<R(b_{1})\Rightarrow 0.42<\lambda \leq 1$, when λ=0.5, it is risk neutral, and the EU-E model is valid (Table 3). For the case of 0.5<λ≤1, decision-makers are risk-seekers, and are willing to take corresponding risks for higher reward. In this case, decision-making based on expected or expected utility value is more consistent with decision-making behaviour. Therefore, it is more likely that $b_{1}$ is chosen.

Many experiments have shown that a majority of subjects have a preference pattern of $a_{1}$ over $b_{1}$; however, it does not prove that the EU-E model is effective, because in reality, $1 million is very important to many people, or many people may not have $1 million in assets at all. Therefore, when faced with the above problem, there is generally a risk-averse tendency. According to our recommended method, the choice is $a_{1}$ for those who are risk-averse. Of course, there will be a small number of people who are risk-seeking and choose $b_{1}$. The results of the above analysis are in agreement with the results of the experiment in which many people chose $a_{1}$, while a few chose $b_{1}$. This further proves the reliability of the proposed method.

**Problem 2**: select $a_{2}$ or $b_{2}$?

Similarly, we have $H(a_{2})=0.3465$, $H(b_{2})=0.3251$, $E(a_{2})=0.11$, $E(b_{2})=0.5$, $R(a_{2})=0.57\lambda -0.22$, $R(b_{2})=1.33\lambda -1$. For any 0≤λ≤1, $R(a_{2})>R(b_{2})$. For problem 2, we must first realise that regardless of whether we choose $a_{2}$ or $b_{2}$, the possibility of zero consequence is almost the same and much greater than the non-zero value. Taking into account the above factors, decision-makers are most likely to show risk-seeking behaviour. According to Table 3, the EU-E model is reasonable, and we should choose $b_{2}$.

Based on the recommended method, we discuss the Allais paradox, and through theoretical analysis, the results obtained by the experiment were reasonably explained.

## 4. Case Analysis and Discussion

### 4.1. Engineering Background

Complex geology will increase the difficulty of obtaining accurate geological information. The uncertainty of geological information increases the risk on a tunnel project; therefore, it is likely to cause geological disasters (landslides, rock bursts, water inrush, etc.) when tunnelling in such areas. For example, southwestern China has the characteristics of abundant groundwater, karst development, and fault fracture zones: tunnel burial depths are generally large, and so on, which causes serious geological hazard risk [44].

**Figure 3:**
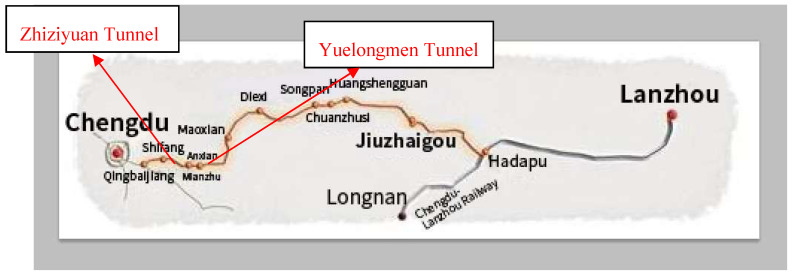
Map of Zhiziyuan tunnel.

The research object is the Zhiziyuan tunnel (the range of tunnel mileage is D2K76+695~D2K90+765) on the Chengdu-Lanzhou railway (Figure 3). The Zhiziyuan tunnel has a length of 14 km, and its maximum burial depth is 680 m. Although Yuelongmen tunnel is longer and is buried deeper, the geological conditions around the Zhiziyuan tunnel are more complicated. The main features are as follows: (1) Karst is developed and widely spread, resulting in serious risk of water inrush during construction. (2) The construction method of Zhiziyuan tunnel is a combination of single-hole and double-hole tunnelling, which is more complex than other tunnel construction environments and suffers greater uncertainties. (3) As the tunnel is close to villages, it is more sensitive to water pollution and ecological damage caused by water inrush. Therefore, a reasonable risk control scheme for water inrush is of great significance for the smooth construction of the Zhiziyuan tunnel. We take the section of D2K81+710~D2K81+770 as an example for analysis. The section is characterized by dolomitic limestone and broken surrounding rock. And the geophysical results show that the fault is wide and the groundwater is rich. In addition, the height of the nearby Jushui river is higher than the height of the tunnel in this area, the river has been cut off due to the construction of the tunnel, and the water has entered the mountain, which has posed a major hidden danger to the tunnel. So it has the representative significance to choose the section.

### 4.2. Engineering Application and Discussion

The mechanism of water inrush accidents is complex, and the mechanism of different types of outburst is quite different. For the karst water inrush disasters, by analysing the existing cases of different inrush water disasters, the relationship between inrush water risk and major factors (water pressure $x_{1}$, formation lithology $x_{2}$, attitude of rocks $x_{3}$, topography and geomorphology $x_{4}$, bad geological conditions $x_{5}$, and the depth from the underground water level $x_{6}$) can be obtained by fitting. Assuming it is $P(A)=0.01x_{1}+0.04x_{2}+0.0003x_{3}+0.02x_{4}^{2}+0.042\sqrt{x_{5}}+0.002\sqrt{x_{1}x_{6}}$ (based on the statistical analysis of the cases of water inrush disasters in several tunnel projects along Chengdu-Lanzhou railway, such as Yuelongmen tunnel, Zhiziyuan tunnel, Pingan tunnel, Maoxian tunnel and so on, the formula can be obtained), which indicates the relationship between the influence factors and the occurrence probability of water inrush. The initial value range of each parameter is as follows: water pressure: 6~8.5 MPa, formation lithology: 0.7~0.86, attitude of rocks: 45~50, topography and geomorphology: 0.3~0.4, bad geological conditions: 6.5~8.3, depth below underground water level: 0.3~0.34.

According to the risk control process constructed in Figure 2, the sensitivity analysis of each parameter is needed, and the parameter that has the greatest impact on the evaluation result can be determined. The probability distribution of P(A) can be calculated by Monte Carlo simulation. The entropy is used to quantify uncertainty measures, and then sensitivity indices of each parameter can be obtained based on entropy sensitivity analysis methods. The related calculations are as follows. H[P(A)]=0.034, $H[P(A)|x_{1}]=0.0166$, $H[P(A)|x_{2}]=0.0295$, $H[P(A)|x_{3}]=0.0329$, $H[P(A)|x_{4}]=0.0330$, $H[P(A)|x_{5}]=0.0243$, $H[P(A)|x_{6}]=0.0335$. Sensitivity indices can be obtained by using the following sensitivity formulae: $\eta (x_{1})=0.5118$, $\eta (x_{2})=0.1324$, $\eta (x_{3})=0.0324$, $\eta (x_{4})=0.0294$, $\eta (x_{5})=0.2853$, and $\eta (x_{6})=0.0147$. The indicators of $x_{1}$, $x_{2}$, $x_{5}$ have a significant influence on the evaluation result for water inrush. Therefore, to improve the efficiency of decision-making, relevant risk control schemes should be formulated around these three indicators.

Suppose we have the following six preliminary risk control schemes (Table 4), and then use Equation (4) to determine which schemes meet the decision-maker’s tolerance costs. According to experienced experts, the occurrence of water inrush will cause a loss of $3.5\times 10^{6}$ RMB. The change in risk under such conditions is $\Delta R_{1}=20.72\times 10^{4}$. The uncertainty measure of inrush water occurrence is H(A)=0.5482. Let α=1, and set tolerance cost $T_{H}^{\prime }=113587$. According to the cost of each scheme in Table 4, the schemes ($a_{1}$, $a_{3}$, $a_{5}$) are in line with the requirements. Therefore, it is necessary to determine the most reasonable scheme according to the multi-attribute decision-making method proposed in this paper.

For the risk control scheme used here in a tunnel engineering project, attributes such as economic cost, time cost, environmental impact, and programme execution effect are generally considered. Therefore, we can make a comprehensive analysis based on the impact of different schemes on attributes when executing the work, and then select the optimal scenario. According to expert opinion, the possible impact of the above three schemes on different attributes can be obtained. Then the benefit-type attributes and the cost-type attributes are normalised separately. The results are shown in Table 5.

The economic cost in the above table is consistent with Table 4, which gives a fixed value. Then the uncertainty measures and expected utility of different attribute information are calculated. The uncertainty measure and expected utility of each scheme are aggregated based on attribute weights ($\omega (c_{1})=0.3$, $\omega (c_{2})=0.2$, $\omega (c_{3})=0.3$, $\omega (c_{4})=0.2$). The comprehensive uncertainty measure and expected utility of each scheme are obtained (Table 6).

From Table 6: $H(a_{1})>H(a_{5})$, $E(a_{1})<E(a_{5})$, $H(a_{3})>H(a_{5})$, $E(a_{3})<E(a_{5})$. If decision-makers are risk-seeking, according to Table 3, $a_{5}(x_{2},x_{5})$ is more reasonable than $a_{1}(x_{1})$ and $a_{3}(x_{1},x_{2})$, so $a_{5}(x_{2},x_{5})$ should be chosen. However, if risk-averse, no direct judgment can be made. We then need to analyse the advantages and disadvantages of different schemes under each attribute. For example, suppose that scheme $a_{1}(x_{1})$ is more reasonable than $a_{3}(x_{1},x_{2})$ under attribute $c_{j}$, then $c_{1j}=1$, $c_{3j}=0$, respectively. Finally, the comprehensive value of each scheme is obtained according to linear weighting and the largest is the decision result. Based on the methods recommended in Table 3, we first compare schemes $a_{1}(x_{1})$ and $a_{5}(x_{2},x_{5})$, and the results are:$c_{11}=0$, $c_{12}=0$, $c_{13}=0$, $c_{14}=0$; $c_{51}=1$, $c_{52}=1$, $c_{53}=1$, $c_{54}=1$. Scheme $a_{5}(x_{2},x_{5})$ is more reasonable. Now we compare scheme $a_{3}(x_{1},x_{2})$ and $a_{5}(x_{2},x_{5})$, and the results are: $c_{31}=0$, $c_{32}=0$, $c_{33}=0$, $c_{34}=1$; $c_{51}=1$, $c_{52}=1$, $c_{53}=1$, $c_{54}=0$. Considering attribute weights: $c(a_{3})=0.2$, $c(a_{5})=0.8$, and $a_{5}(x_{2},x_{5})$ should be chosen. Therefore, whether decision-makers are risk-averse, or risk-seeking, scheme $a_{5}(x_{2},x_{5})$ is the more reasonable choice.

## 5. Conclusions

Reasonable risk control measures are an effective way in which to reduce geological disasters in underground engineering operations. As geological uncertainty is the main feature of geotechnical engineering, according to the latest definition of risk, it can be known that uncertainty exerts a significant impact on risk control. Therefore, a reasonable assessment and control of uncertainty is an important part of risk control. This paper focuses on the problem of uncertainty evaluation and control. The main conclusions are as follows:

(1) Through case analysis, possible defects in the existing entropy-hazard decision model in engineering application are discussed, and an improved model of uncertainty evaluation and control is proposed.
(2) Tolerance cost $T_{H}^{\prime }$ is an important factor used to screen plans to improve the efficiency of decision-making. Through analysis, it is found that there are deficiencies in the existing calculation method, and a new calculation model is proposed.
(3) The existing expected utility-entropy model is only valid under certain conditions, and there are limitations to its application. Multiple-attribute decision making problems are classified based on factors such as attitude to risk, uncertainty measures, expected utility, variance, etc., and corresponding decision-making methods are proposed according to different decision types.
(4) By analysing different decision-making issues, it is found that the attitude to risk of the decision-makers exerts an important influence on the decision-making results; attitudes to risk will be influenced by the decision-maker’s own experience and the problem itself. Based on this idea, the well-known Allais paradox is reasonably explained by use of the proposed methods.
(5) Taking the Zhiziyuan tunnel as a research object, the method is applied to a real engineering task. The application results show that the proposed method is effective with regard to decision-making about geological disaster risk control schemes, and that it can remedy the deficiencies in the existing method.

## Figures and Tables

**Table 1:** The combination of uncertainty and hazard.

Serial Number	U	$R_{1}$	Serial Number	U	$R_{1}$
1	Small	Small	3	Large	Large
2	Small	Large	4	Large	Small

**Table 2:** Different decision problems and their solutions.

Condition 1	Condition 2	Condition 3	Risk Attitude	Suggested Methods
H(a)=H(b)	E(a)=E(b)	R(a)=R(b)	Risk-averse	Variance
			Risk-neutral	Variance
			Risk-seeking	If $pa_{\max}>qb_{\max}$, then choose *a*, otherwise choose *b*
	E(a)<E(b) (E(a)>E(b))	R(a)>R(b) (R(a)<R(b))	Risk-averse	If $la_{\min}>mb_{\min}$, choose *a*, otherwise choose *b*
			Risk-neutral	EU-E model
			Risk-seeking	EU-E model
H(a)>H(b) or (H(a)<H(b))	E(a)=E(b)	R(a)>R(b) (R(a)<R(b))	Risk-averse	Variance
			Risk-neutral	Variance
			Risk-seeking	If $pa_{\max}<qb_{\max}$, then choose *b*, otherwise choose *a*
	E(a)>E(b) (E(a)<E(b))	R(a)=R(b)	Risk-averse	Variance
			Risk-neutral	Variance
		R(a)>R(b)	Risk-averse	Variance
			Risk-neutral	There is no uniform criterion, the recommendation is similar to that when risk-averse
			Risk-seeking	If $pa_{\max}<qb_{\max}$, then choose *b*, otherwise choose *a*
		R(a)<R(b)	Risk-averse	EU-E model
			Risk-neutral	EU-E model
	E(a)<E(b) (E(a)>E(b))	R(a)>R(b) (R(a)<R(b))	Risk-averse	If $la_{\min}>mb_{\min}$, choose *a*, otherwise choose *b*
			Risk-neutral	There is no uniform criterion, the recommendation is similar to that when risk-averse
			Risk pursuit	EU or EU-E model

**Table 3:** Allais paradox.

Schemes	Probability	Consequence	Probability	Consequence	Probability	Consequence
$a_{1}$	1	1 (million)	-	-	-	-
$b_{1}$	0.01	0	0.89	1 (million)	0.1	5 (million)
$a_{2}$	0.89	0	0.11	1 (million)	-	-
$b_{2}$	0.9	0	0.1	5 (million)	-	-

**Table 4:** Initial risk control schemes.

Schemes	Expense	Schemes	Expense
$a_{1}(x_{1})$	103,000	$a_{4}(x_{1},x_{5})$	120,000
$a_{2}(x_{1})$	115,000	$a_{5}(x_{2},x_{5})$	98,000
$a_{3}(x_{1},x_{2})$	110,000	$a_{6}(x_{1},x_{2},x_{5})$	135,000

**Table 5:** Initial attribute information (normalised data).

	Attributes	C_1_ (Economic Cost)	C_2_ (Time Cost)	C_3_ (Environment Impact)	C_4_ (Execution Effect)
Schemes					
$a_{1}(x_{1})$		0.5829	(0.5, 30%; 0.75, 60%; 1, 10%)	(0.5385, 20%; 0.3846, 50%; 0.2308, 30%)	(0, 20%; 0.3429, 40%; 0.7143, 40%)
$a_{3}(x_{1},x_{2})$		0	(0, 15%; 0.25, 62%; 0.5, 23%)	(0.3846, 40%; 0.1538, 50%; 0, 10%)	(0.5714, 30%; 0.7143, 50%; 1, 20%)
$a_{5}(x_{2},x_{5})$		1	(0.75, 25%; 0.875, 70%; 1, 5%)	(1, 10%, 0.8462, 50%; 0.6923, 40%)	(0.2857, 28%; 0.5714, 52%; 0.8571, 20%)

**Table 6:** Uncertainty measure and expected utility.

	Attributes		C_1_ (Economic Cost)	C_2_ (Time Cost)	C_3_ (Environment Impact)	C_4_ (Execution Effect)	Aggregation
Schemes							
$a_{1}(x_{1})$		Entropy	0	0.8979	1.0297	1.0549	0.6995
		EU	0.5829	0.7	0.3692	0.4229	0.5102
$a_{3}(x_{1},x_{2})$		Entropy	0	0.9190	0.9433	1.0297	0.6727
		EU	0	0.27	0.2307	0.7286	0.2689
$a_{5}(x_{2},x_{5})$		Entropy	0	0.7460	0.9433	1.0184	0.6359
		EU	1	0.85	0.8	0.5485	0.8197
